# Initial Experience with Transarterial Chemoembolization in Metastatic Cutaneous Melanoma Patients with Hepatic Progression on Immune Checkpoint Inhibitors

**DOI:** 10.1007/s00270-025-04218-0

**Published:** 2025-10-27

**Authors:** Timo Alexander Auer, Max Schlaak, Gabor Dobos, Lynn Jeanette Savic, Florian Nima Fleckenstein, Thomas Eigentler, Federico Collettini

**Affiliations:** 1 Department of Radiology, Charité University Medicine Berlin, Charité Campus Mitte, Charitéplatz 1, Berlin, 10117 Germany; 2 Department of Dermatology, Berlin Institute of Health, Humboldt-Universität Zu Berlin, Berlin, Germany; 3 Berlin Institute of Health (BIH), Anna-Louisa-Karsch 2, 10178 Berlin, Germany

**Keywords:** Melanoma, TACE, Chemoembolization, Liver metastases, ICI

## Abstract

**Objectives:**

To assess the safety and efficacy of performing degradable starch microspheres transarterial chemoembolization (DSM-TACE) in metastatic melanoma (MM) patients with progressive liver metastases following immune checkpoint inhibitors (ICI) treatment.

**Material and Methods:**

This case series evaluates 10 adult patients with MM who exhibited hepatic progression after an initial response to ICI therapy and underwent DSM-TACE without discontinuation of systemic checkpoint inhibitor therapy from January 2023 to September 2024. Tumor response was assessed using RECIST 1.1 criteria. The primary outcome measure of the study was the best overall response rate. Secondary outcome measures included local tumor control, progression-free survival, and overall survival. Adverse events were monitored and recorded.

**Results:**

Ten melanoma patients (median age: 69.5 years; six females) who underwent DSM-TACE due to hepatic progression following an initial response to ICI therapy were included for safety analysis. Imaging data were unavailable for two patients, who were therefore excluded from further analyses. Best overall response rate was 87.5% (CR in two patients, PR in five patients, and SD in one patient). All treated lesions displayed sustained local tumor control throughout the follow-up. Six patients (75%) experienced the occurrence of new intra- or extrahepatic metastases. The median PFS was 8.5 months. Median OS was 12 months. Severe or life-threatening (grade 3–4) AEs associated with the combined treatment were reported in 40% (4/10—pseudoaneurysm at the puncture side, immune-related pancreatitis, anthracnosis, and colitis).

Cocnclusion.

Incorporating locoregional therapies, such as DSM-TACE, in patients with liver metastases from cutaneous melanoma undergoing ICI treatment is feasible, safe, and effective in achieving local disease control, potentially extending PFS and OS.

## Introduction

Melanoma patients with liver metastases treated with immune checkpoint inhibitors (ICIs) exhibit lower response rates and shorter progression-free survival compared to those without liver involvement [1]. Liver metastases have been shown to exploit the liver’s unique immunosuppressive microenvironment to evade immune surveillance, thereby diminishing the effectiveness of systemic ICIs not only locally but also at distant, extrahepatic metastatic sites [2, 3]. To address resistance associated with liver metastases, novel therapeutic approaches are being explored [4]. Liver-directed therapies, such as transarterial chemoembolization (TACE), hold promise not only for directly targeting the liver metastases but also for modulating the liver’s immune microenvironment [5]. TACE may disrupt the immune-suppressive milieu typically associated with liver metastases, enhancing the efficacy of ICI [5]. Herein, we report on our initial experience with a cohort of ten patients with acquired resistance to ICI therapy who underwent TACE due to hepatic progression. The aim of this study was to assess the safety and the potential clinical benefits of performing TACE in metastatic melanoma patients with progressive liver metastases following ICI treatment.

## Methods

This case series included patients with metastatic melanoma who exhibited hepatic progression after an initial response to ICI therapy (anti-PD1±Ipilimumab) and underwent TACE without discontinuation of systemic ICI therapy. This study was approved by the institutional review board (EA4/052/13). The requirement for informed consent was waived due to the retrospective study design.

### Treatments

At the time of hepatic progression, patients were receiving either intravenous anti-PD1 monotherapy with nivolumab (*n* = 6; 480 mg every 4 weeks) or pembrolizumab (*n* = 1; 400 mg every 6 weeks), or nivolumab combined with ipilimumab (*n* = 3; 1 mg/Kg nivolumab + 3 mg/Kg ipilimumab every three weeks for four cycles, subsequently nivolumab 480 mg every 4 weeks). Systemic ICI was continued using the same agent and dosage during and after the DSM-TACE cycles, until either further disease progression unmanageable by DSM-TACE or the occurrence of unacceptable toxicity, respectively. TACE procedures were performed in the intervals between intravenous ICI infusions using a suspension of 450 mg of degradable starch microspheres (EmboCept® S DSM 50 µm, Magle PharmaCept) mixed with cisplatin (50–100 mg) as previously described [6, 7]. Briefly, after performing a selective angiogram of the coeliac axis to identify tumor-feeding arteries, a coaxial microcatheter was advanced into the relevant branches of the hepatic artery. DSM-TACE was carried out either super-selectively or non-selectively, based on tumor number and location. Once the microcatheter was correctly positioned, a suspension of 450 mg/7.5 ml of DSM (EmboCept® S DSM 50 µm, Magle PharmaCept) was mixed with 50–100 mg of cisplatin and 17.5 ml of contrast agent according to manufacturer guidelines and slowly infused under fluoroscopy. For lobar embolization, the procedure continued until a "tree-in-the-winter" angiographic appearance was achieved, indicating blockage of small tumor vessels while maintaining flow in major hepatic arteries. Patients were scheduled to receive three consecutive TACE procedures with 6-week intervals. Additional TACE procedures were performed on demand in case of residual viable disease or further hepatic progression.

### Efficacy and Safety evaluation

Tumor response was assessed every two to three months by computed tomography or magnetic resonance imaging. The primary outcome measure of the study was the best overall response rate (bORR), defined as the best response recorded from the start of the combined treatment until the last follow-up visit, according to RECIST 1.1 criteria [8]. Secondary outcome measures included local tumor control (LTC), progression-free survival (PFS), and overall survival (OS). Adverse events (AEs) were monitored and recorded during and after each treatment, and at all follow-up visits. AEs were graded per patient according to the Common Terminology Criteria for Adverse Events (CTCAE) version 5.

### Statistics

Statistical analysis was conducted using the XLSTAT statistical and data analysis solution (Addinsoft). Continuous variables are displayed as median and interquartile range (IQR), whereas categorical variables are displayed as frequencies and percentages. Due to the small sample size, the statistical analysis was limited to descriptive statistics.

## Results

### Patients

From January 2023 to September 2024, ten melanoma patients (median age: 69.5 years, six females) with new hepatic metastases on ICI underwent TACE of the liver metastases. Patient baseline characteristics are presented in Table 1. Patients were on systemic ICI therapy for a median time of 42 months (IQR 22.3–56.3) before experiencing acquired resistance with hepatic progression. At the time of the first TACE procedure, eight out of ten patients had multifocal hepatic disease with a median tumor count of six (IQR 3.0–10). The median diameter of the largest liver metastasis was 32 mm (IQR 18.5–45.8). Patients underwent a median of three TACE procedures (IQR 3.0–3.8). Five patients had concomitant, limited extrahepatic disease.

**Table 1:** Patients baseline characteristics

Subject	Sex	Age	Tumor stage (UICC)	Extrahepatic metastases	Extrahepatic response	KPS	Mutation status	AE ICI
1	female	63	pT4apN2ccM1c	No	–	100	BRAF WT	IA-pancreatitis
2	male	47	pT3apN3cM1c	Yes (LN, bone)	no	100	BRAF WT	IA-anhrakosis
3	male	53	pT4bpN1cM1c	Yes (Brain)	yes (brain)	100	BRAF WT	IA-colitis
4	female	72	pT4bN0M1c	No	–	90	BRAF K601N	No
5	female	67	pTXc pN2c cM0	Pulmonal, LN,	Yes (pulmo, LN)	100	BRAF WT	No
6	female	60	pT0N2bM1c	Yes (Bone, lung)	no	100	BRAF WT	No
7	female	86	pT3bN0M1c	No	–	90	BRAF WT	No
8	male	83	pT3apN1bpM1c	No	–	100	BRAF WT	No
9	female	72	pT4bpN0sn0/4cM1c	Yes (LN, brain)	no	100	BRAF WT	No
10	male	81	pT3apN1a(sn)M1c	No	–	100	BRAF WT	No

*UICC* Union internationale contre le cancer*, LN *Lymph nodes, *KPS* Karnofsky performance status scale, *AE*: Adverse events, *ICI* Immune checkpoint inhibitors, *IA* Immune associated

### Safety and Efficacy

**Fig. 1:**
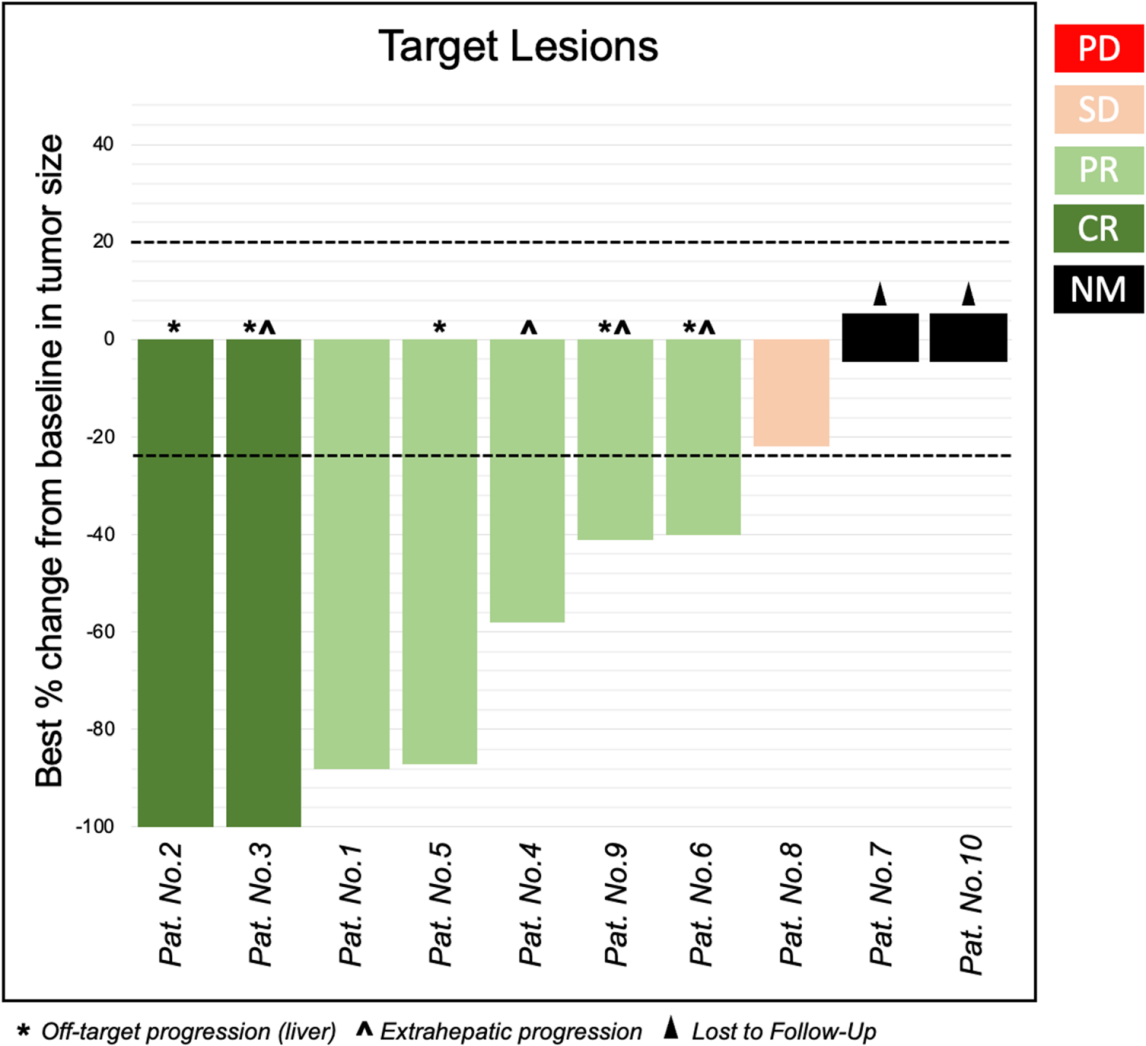
Waterfall plot showing the bORR *PD: Progressive disease; SD: Stable disease; PR: Partial response; CR: Complete response; NM: Not measurable

**Fig. 2:**
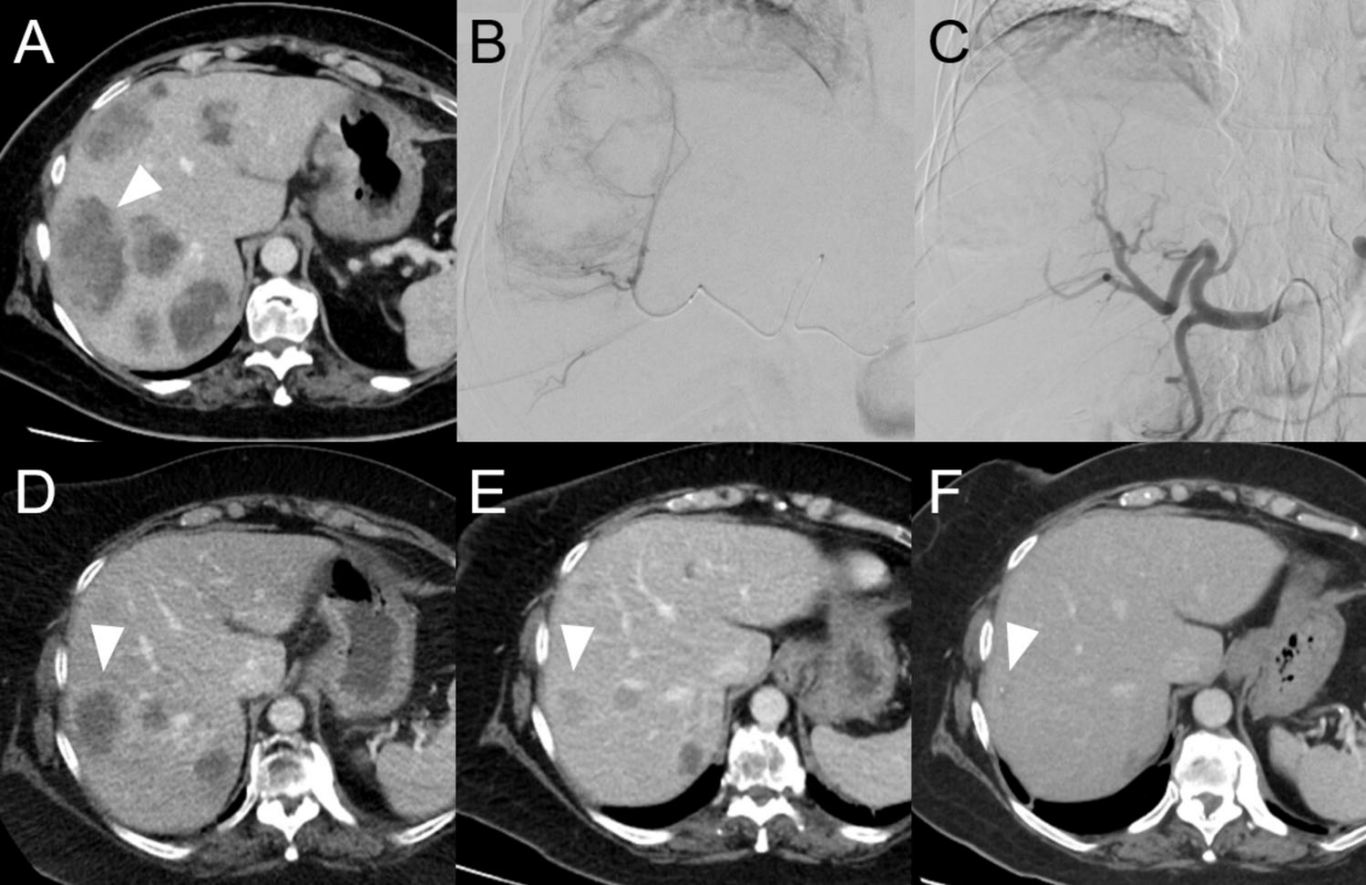
63-year-old female patient (No. 1) experiencing hepatic disease progression under anti-PD1 therapy. **A** Baseline axial contrast-enhanced computed tomography showing multiple bilobar liver metastases up to 8 cm in diameter (the largest lesion is marked with a white arrowhead). **B** First TACE procedure performed with the microcatheter (black arrowhead) positioned in the right hepatic artery. **C** Digital subtraction angiography performed with the guiding catheter in the celiac trunc right after the TACE procedure with no visible tumor blush. **D–F** Follow-up imaging at 3- **D**, 6- **E**, and 18 months after the first TACE **F**

At the time of data cutoff, six patients were alive (four patients died—causes of death: 3 × unknown outside the hospital, 1 × intracranial bleeding), three of them without progressive disease. The median follow-up time was 12 months (IQR 6.8–14.3). Imaging data were available for eight patients. bORR was 87.5% (CR in two patients, PR in five patients, and SD in one patient). All treated lesions displayed sustained local tumor control throughout the follow-up. Interestingly enough, among the five patients with extrahepatic metastases, two demonstrated a clear reduction in extrahepatic lesions that had previously been progressing under ICI therapy, following the administration of DSM-TACE (Fig. 1). The median tumor burden reduction was 72% (IQR 40.8–91.0). The waterfall plot in Fig. 2 shows the best percentage change from baseline in the sum of the diameters of the treated lesions. Six patients (75%) experienced the occurrence of new intra- or extrahepatic metastases. The median PFS was 8.5 (IQR 5.5–9.0) months. Median OS was 12 months (IQR 6.8–14.3). Severe or life-threatening (grade 3–4) AEs associated with the combined treatment were reported in 40% (4/10—pseudoaneurysm at the puncture site, immune-related pancreatitis, anthracnosis, and colitis, all grade 3). Mild-to-moderate AEs were observed in 20% (2/10) of patients (Nausea, grade 2 AE).

## Discussion

Our findings indicate that conducting repetitive DSM-TACE procedures while continuing immunotherapy is both feasible and safe. We specifically chose DSM-TACE because this technique offers a refined and effective means of managing patients with multifocal hepatic disease and high intrahepatic tumor burden. Its favorable safety profile allows for comprehensive tumor control without significantly compromising overall liver function [6].

**Fig. 3:**
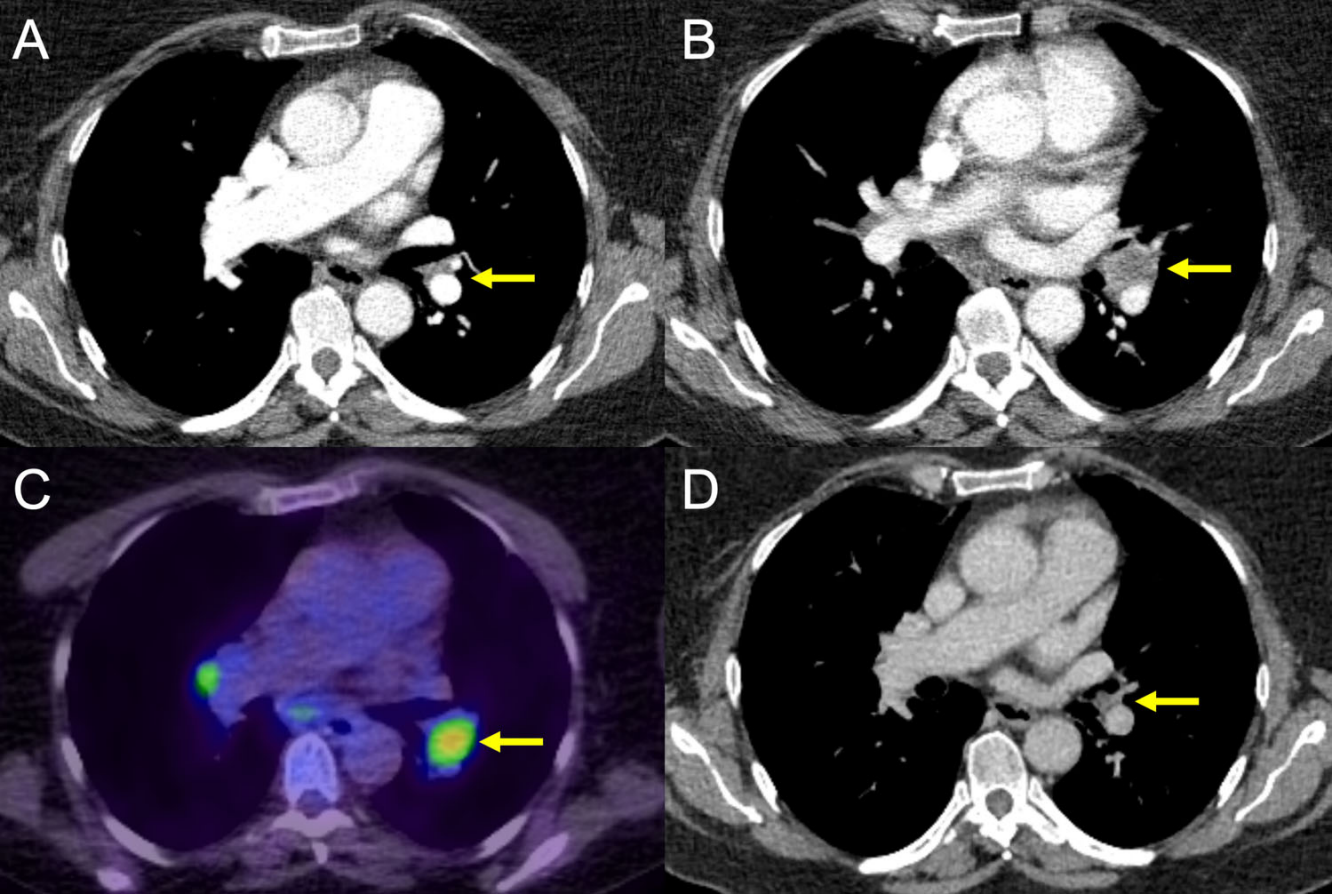
67-year-old female patient (No. 5) experiencing hepatic and mediastinal lymph node disease progression under anti-PD1 therapy. **A** shows an axial contrast-enhanced CT (Angio-CT) of the thorax from March 2023 with no evidence of definitive mediastinal or parahilar lymph node metastases. **B** shows an axial contrast-enhanced CT of the thorax from the follow-up in August 2023 with clear evidence of mediastinal and parahilar lymph node metastases. **C** shows the corresponding PET-CT. **D** shows the follow-up CT in May 2025 after combined ICI and TACE therapy showing a complete response of the extrahepatic metastases, indicating an abscopal effect (the yellow arrows indicate the target lesion in each image)

While two patients experienced pain and nausea after DSM-TACE (grade 2), which could be treated with intravenous metamizol, the remaining patients tolerated the procedure well. The only DSM-TACE-related grade 3 AE was a pseudoaneurysm of the common femoral artery, which was successfully treated with thrombin injection. Imaging results revealed a convincing response to the combined treatment, with a median tumor burden reduction of 72% (IQR 40.8–91.0) (Fig. 3).

Notably, in all eight patients with available follow-up imaging, none of the treated liver metastases exhibited local progression over time. These findings demonstrate that following the development of acquired resistance, combining systemic ICI therapy with DSM-TACE can achieve a renewed local tumor response, potentially leading to complete hepatic remission. The median PFS (8.5 months) and median OS (12 months) observed in this study compare favorably with previously reported data in the literature. In a recent study by Goldinger et al., patients experiencing tumor progression under anti-PD1 therapy displayed a median PFS of 2.6 months and a median OS of 7.1 months [9].

Several studies have investigated the immunomodulatory effects of TACE and provided rationales for combining TACE with ICI. TACE has been shown to modulate the tumor microenvironment by inducing immunogenic cell death, which enhances the presentation of tumor antigens and stimulates an anti-tumor immune response [10]. This process can upregulate immune checkpoints, suggesting a potential benefit from combining TACE with ICI [5]. A study by Marinelli et al. demonstrated that TACE combined with PD1 blockade could be safely administered and may lead to significant delays in tumor progression in selected patients with hepatocellular carcinoma [11]. Although we did not conduct repeat biopsies to assess the immunologic response following TACE and therefore cannot directly confirm a synergistic interaction between systemic ICI and TACE, the substantial disease response observed herein and the fact that, in two patients, extrahepatic lesions that had previously been progressing under ICI therapy showed a clear reduction in size following the administration of DSM-TACE highlights the need for further research to investigate a potential synergy between systemic ICI and TACE in patients with cutaneous melanoma metastatic to the liver. A potential synergistic effect is further supported by the fact that the very limited data available in the literature on the use of TACE as a stand-alone therapy for the treatment of hepatic metastases from cutaneous melanoma are, for the most part, unconvincing [12, 13]. In future studies combining TACE with systemic therapies, it would also be reasonable to explore the potential of combination therapies incorporating a VEGF inhibitor, as demonstrated in recent phase 3 trials investigating combined treatment strategies for hepatocellular carcinoma [14].

## Limitations

The study has clear limitations that should be briefly addressed. Firstly, the very small patient sample and the retrospective design restrict the generalizability of the findings. Additionally, the absence of pre- and posttreatment biopsies prevents us from directly confirming a synergistic interaction between systemic ICI and TACE.

## Conclusion

In conclusion, despite the clear limitations of a small patient cohort and short follow-up period, the findings of this case series suggest that incorporating locoregional therapy, such as TACE, in patients with liver metastases from melanoma undergoing ICI treatment is feasible, safe, and effective in achieving local disease control, potentially extending PFS and OS. Translational studies exploring the potential synergistic interaction between TACE and ICI are warranted.
